# Saliency determines the integration of contextual information into stimulus–response episodes

**DOI:** 10.3758/s13414-021-02428-5

**Published:** 2022-01-19

**Authors:** Ruyi Qiu, Malte Möller, Iring Koch, Susanne Mayr

**Affiliations:** 1 Chair of Psychology and Human-Machine Interaction, University of Passau, 94032 Passau, Germany; 2 Department of Cognitive and Experimental Psychology, RWTH Aachen University, 52056 Aachen, Germany

**Keywords:** Context, Saliency, Stimulus–response episode, Binding

## Abstract

When humans perform a task, it has been shown that elements of this task, like stimulus (e.g., target and distractor) and response, are bound together into a common episodic representation called *stimulus–response episode* (or *event file*). Recently, the context, a completely task-irrelevant stimulus, was found to be integrated into an episode as well. However, instead of being bound directly with the response in a binary fashion, the context modulates the binary binding between the distractor and response. This finding raises the questions of whether the context can also enter into a binary binding with the response, and if so, what determines the way of its integration. In order to resolve these questions, saliency of the context was manipulated in three experiments by changing the loudness (Experiment 1) and emotional valence (Experiment 2A and 2B) of the context. All experiments implemented the four-alternative auditory negative priming paradigm introduced by Mayr and Buchner (2006, *Journal of Experimental Psychology: Human Perception and Performance, 32*[4], 932–943). Results showed that the integration of context changed as a function of its saliency level. Specifically, the context of low saliency was not bound at all, the context of moderate saliency modulated the binary binding between the distractor and response, whereas the context of high saliency entered into a binary binding with the response. The current results extend a previous finding by Hommel (2004, *Trends in Cognitive Sciences, 8*[11], 494–500) that there is a saliency threshold which determines whether a stimulus is bound or not, by suggesting that a second threshold determines the specific structure (i.e., binary vs. configural) of the resulting binding.

While we perceive a given stimulus as a unit, features of the stimulus (e.g., color and shape of an object or pitch and loudness of a sound) are coded in a distributed fashion in the brain (e.g., Seymour et al., 2009; Stecker et al., 2005). This raises the so-called binding problem, which has stimulated a long and still ongoing research interest (for reviews, see Feldman, 2013; Treisman, 1996). Kahneman et al. (1992) were among the first to investigate feature binding and proposed that an episodic trace was formed to store the features and their relations when processing a stimulus. Similarly, it has been shown that stimuli and responses can also be integrated into a common episodic representation called stimulus–response episode (or event file, for overviews, see Hommel, 2004; also see the Binding and Retrieval in Action Control [BRAC] framework; Frings et al., 2020). It has been proposed that reencountering one of the elements of an episode will retrieve the whole episode, which may facilitate or impair responding, depending on whether the retrieved episode is compatible with the current processing demands or not.

Binding and retrieval of stimulus–response episodes is assumed to be a general mechanism of human information processing which underlies several empirical phenomena (Frings et al., 2020), the one most relevant to the present purpose is the *negative priming effect*. In a typical negative priming task, participants need to respond to a target stimulus and simultaneously ignore a distractor stimulus. When the distractor stimulus of a first presentation (i.e., the *prime*) reappears as the target in the following presentation (i.e., the *probe*) in so-called *ignored repetition* trials, responses are slowed down and sometimes more error prone as compared with trials without any stimulus repetition (so-called *control* trials). The impaired responding in ignored repetition as compared with control trials denotes the *negative priming effect* (Neill, 1977; Tipper, 1985).^1^ Based on the instance theory of automatization (Logan, 1988), Neill and Valdes (1992) proposed that by withholding a response to the distractor stimulus in the prime, this stimulus is associated with the so-called *do-not-respond* tag. When the stimulus is repeated as the target in the probe, the do-not-respond tag is retrieved and conflicts with the need to respond to this stimulus in the probe, thus impairing the response speed and/or accuracy.

Alternatively, and in line with BRAC framework of binding and retrieval of stimulus–response episodes, the executed prime response is bound with the other elements of the prime trial (e.g., target and distractor stimuli). Therefore, reencountering the prime distractor stimulus in the probe will retrieve the prime episode, including the prime response. This is exactly what Mayr and Buchner (2006) found. In their experiment, the prime response was always different from the correct probe response. This implies that retrieving the prime response in ignored repetition trials should impair probe responding, thereby leading to the negative priming effect (for a similar explanation, see Rothermund et al., 2005). Mayr and Buchner (2006) used a four-alternative identification task, in which each stimulus was assigned to a unique response key. This allowed the authors to analyze the frequencies of the different probe response types. Specifically, a probe response could be categorized as either a correct response, an erroneous response with the key assigned to the probe distractor, an erroneous execution of the prime response, or an erroneous response with the remaining response option. Results showed an increased probability of committing errors with the former prime response in the ignored repetition as compared with the control condition. Since only elements that were bound together can be retrieved by the repetition of one of them, the effective retrieval of the prime response by the repetition of the prime distractor stimulus indicates that a binding was formed between these elements. The increased probability of committing prime response errors induced by the repetition of the prime stimulus has been coined as the *prime-response retrieval effect*, which is an unambiguous indicator of stimulus–response binding (Frings et al., 2015; Mayr et al., 2018).

In the present article, we adopted the negative priming paradigm and the analysis of the prime-response retrieval effect as a tool to investigate the mechanisms of stimulus–response binding with respect to the role of context in the integration of stimulus–response episodes.

## The role of context in binding and retrieval of stimulus–response episodes

Context can act as an effective retrieval cue. For example, there is consistent evidence from the memory literature showing that the similarity of contextual information between the encoding and testing phases favors successful retrieval (for a review, see Smith & Vela, 2001). Given that stimulus–response episodes are stored in memory and are retrieved from memory, the context may also play an important role in the binding and retrieval of stimulus–response episodes. For instance, Frings and Rothermund (2017) tested the integration of contextual visual features (e.g., color) using the distractor-response binding paradigm, examining the effect of the relationship between distractor repetition and response repetition on performance. In accordance with the rules of figure–ground segmentation (for a review, see Wagemans et al., 2012), they found that features belonging to the figure region (e.g., a confined area on the screen) were bound with the response whereas features belonging to the background did not result in measurable stimulus–response binding effects.

In a recent negative priming study of Mayr et al. (2018), the integration of context into stimulus–response episodes was investigated by means of the before-mentioned four-alternative identification task in the auditory modality (Mayr & Buchner, 2006). The context was a sine tone that was presented together with pairs of task-relevant stimuli (i.e., target and distractor sounds), but it was completely task-irrelevant (i.e., in contrast to targets and distractors, context stimuli were not assigned to a response throughout the experiment). The context tone could be repeated or changed between prime and probe presentations. Results showed no significant prime-response retrieval effect induced by context repetition per se. However, when the context was repeated, the prime-response retrieval effect induced by the repetition of the prime distractor stimulus was significantly larger than when the context was changed (for a similar finding for the distractor-response binding effect with task selection criterion as context, please see Frings et al., 2017). The combined pattern of results—no prime-response retrieval effect induced by context repetition alone on the one hand, and contextual modulation of the prime-response retrieval effect induced by the repetition of the prime distractor stimulus on the other hand—suggests that the context is not bound directly with the response, but that it enters into some kind of higher-order binding with the distractor stimulus and the response (Hommel, 1998).

Evidence of binding among context, stimulus, and response as found by Mayr et al. (2018) fits well into the binding structures proposed by Moeller et al. (2016). The latter authors distinguished between a unitary structure integrating an individual feature and the response (a so-called *binary binding*) and an integration among several features and the response, referred to as *configural binding*. Accordingly, the integration of context found in Mayr et al. (2018) can be categorized as a configural binding—that is, the context and distractor form a compound which is bound with the response. Mayr et al. (2018) replicated the evidence of configural binding of the context in a second experiment. However, it remains an open question whether context is limited to be involved in configural binding structures or it can also enter into a binary binding with the response. The main purpose of the current study was to investigate whether context can be part of different binding structures (either binary or configural) and to pinpoint a factor that determines its binding structure.

## Evidence from learning research: The role of context saliency

The impact of context on behavior has been intensively investigated in the learning literature. Interestingly, contextual information also plays various roles in learned behavior (for reviews, see Bouton, 2010; Bouton & Todd, 2014; Pearce & Bouton, 2001). In some cases, the context directly elicits behavior in a similar way as other stimuli. For example, rats established a contextual fear (indicated by behavior like freezing or avoidance) of the Skinner box or chamber where they were shocked (e.g., Bouton, 1984; Fanselow, 1980). In other cases, the context modulates the association between stimulus and behavior. For example, exposure to the same context where the rats were shocked augmented the rats’ fear of the conditioned stimulus after the extinction manipulation (e.g., Bouton, 1984; Bouton & King, 1986). Saliency has been proposed as one factor that determines the role of context in learning (Bouton, 2010). Saliency is a stimulus property that reveals how conspicuous the stimulus is when compared with its surroundings (Kayser et al., 2005). Evidence shows that stimuli of relatively low saliency rather modulate learned associations than directly elicit behavior, whereas highly salient stimuli tend to be directly associated with the behavior (e.g., Goddard & Holland, 1996; Holland, 1989; Holland & Haas, 1993).

Saliency also plays a role in binding and retrieval of stimulus–response episodes. For example, the level of saliency was found to determine whether a stimulus is integrated into a stimulus–response episode or not (Dutzi & Hommel, 2009; Hommel, 2004). Moreover, Moeller et al. (2016) found that distinguishable features were involved in binary bindings with a response, whereas features that were hard to separate from each other were involved in configural bindings. If saliency increases the distinguishability of a feature, it is possible that features of higher saliency are more likely to be directly bound with a response. In contrast, less salient features may be more likely to be involved in configural bindings or even not integrated into a stimulus–response episode at all. With respect to auditory perception, loudness was found to be positively correlated with the perceived saliency level (Kayser et al., 2005). In Mayr et al. (2018), the saliency of the context might have led to a configural binding because the context tones were approximately as loud as the target and distractor sound pair. Presumably, these context tones were not perceived as of high saliency, and thus the context only modulated the binding between distractor and response instead of being directly bound with the response. The present study aimed to test whether saliency influences the binding of contextual information, and to specify under which saliency conditions the context (1) is not at all integrated into a stimulus–response episode, (2) is involved in a configural binding, or (3) is involved in a binary binding.

## The current study

The current study adopted the paradigm used by Mayr et al. (2018) and manipulated the saliency level of the context. In Experiment 1, saliency was manipulated by changing the loudness of context tones. Specifically, context tones were softer than the sound pair in the *low-saliency condition*, they were approximately as loud as the sound pair in the *moderate-saliency condition*, and they were louder than the sound pair in the *high-saliency condition*. In addition to perceptual properties, information carried by a stimulus can also influence its saliency (e.g., endowing the stimulus with different identity relevance can change the social saliency; Sui et al., 2012). Therefore, in Experiment 2A, which served as a conceptual replication of Experiment 1, emotionally neutral and negative information (in other words, emotional valence) was used to manipulate the saliency of the contextual stimulus. To further confirm the reliability of the findings in Experiment 2A, Experiment 2B was conducted as a full replication of Experiment 2A.

If saliency modulates the integration of context, low-saliency contexts, even if easily perceivable, may not be integrated at all (Hommel, 2004). Thus, repeating the low-saliency contexts should neither retrieve the prime response directly nor facilitate the retrieval of the prime response induced by the repetition of the prime distractor stimulus. As for moderate-saliency contexts, a replication of the findings by Mayr et al. (2018) is expected: The contextual stimulus should be involved in a configural binding—that is, a larger prime-response retrieval effect induced by the repetition of the prime distractor stimulus should be found when the context is also repeated than when it is changed. High-saliency contexts, on the other hand, may be bound directly with the response. This binary binding should be indicated by a significant prime-response retrieval effect due to the repetition of the context per se.

## Experiment 1

### Method

#### Participants

Of the 134 participants who took part in the experiment, data of 28 participants had to be excluded. Of the excluded participants, 24 were tested on a computer with an incorrectly set system volume, and three quit due to keyboard malfunction. The remaining four participants had excessive error rates (>.50) in the ignored repetition and control conditions (as compared with an average error rate of around .09), suggesting either inability or unwillingness to follow the instructions. The resulting sample consisted of 106 adults (84 females), most of whom were students at the University of Passau. They ranged in age from 18 to 32 years (*M* = 22, *SD* = 2.56). Participants either were paid 12 euros or received course credit for their participation. This and the following experiment were conducted in accordance with the ethical guidelines of the German Psychological Association (DGPs) and the Professional Association of German Psychologists (Deutsche Gesellschaft für Psychologie, 2016) and with the 1964 Declaration of Helsinki.

#### Materials

Four 300-ms environmental sounds (frog, piano, drum, and bell) were used as stimuli. Participants heard sounds via headphones (DT110, Beyerdynamic GmbH & Co. KG, Heilbronn, Germany) that were plugged directly into the computer that controlled the experiment. All sounds had an average loudness of approximately 71 dB(A) SPL. Loudness was measured using the NIOSH (2016) app on a cellphone (iPhone 8, Apple Inc., Cupertino, CA, USA) equipped with an external microphone (iMM-6 iDevice Calibrated Measurement Microphone, Dayton Audio, Springboro, USA) while the sounds were played at one side of the headphone. LiveCode (LiveCode 9.5, Runtime Revolution Ltd., Edinburgh, Scotland) was used to program and run the experiment.

In each presentation, a 20-ms metronome click was first played either to the left ear or right ear, indicating the side the participants should pay attention to. After a 500-ms interval, the to-be-attended sound (i.e., target) was played on this side, and a to-be-ignored sound (i.e., distractor) was played simultaneously on the other side. Participants were required to respond to the target sound by pressing an assigned response key, and to ignore the distractor sound. The response keys were four vertically aligned keys (“9,” “6,” “3,” “,”) on the number pad of a keyboard, assigned to the sounds of “frog”, “piano”, “drum”, and “bell”, respectively. Half of the participants were instructed to use their middle and index fingers of their right hands to press the two distal keys, and the middle and index fingers of their left hands to press the two proximal keys. This arrangement was reversed for the remaining participants.

A context tone was played together with the sound pair. The context was a sine tone of either 300 Hz or 700 Hz, also lasting for 300 ms (including 10-ms attack and decay intervals). Context tones were easily discernable not only from the stimulus sounds, but also from each other. Context tones were played simultaneously to both ears creating the impression to come from a central location. The saliency level of context tones was classified as *low*, *moderate*, or *high*, depending on their loudness. In the low-saliency condition, the context tones were softer than the sound pair but still audible (approximately 58 dB(A) SPL); in the moderate-saliency condition, the tones were approximately as loud as the sound pair (about 72 dB(A) SPL); in the high-saliency condition, the tones were louder than the sound pair (approximately 76 dB(A) SPL). When added to the sound pair presentation, context tones of low saliency only slightly increased the overall loudness (approximately 0.5 dB(A) SPL), the moderately salient context tones increased the overall loudness somewhat more (<2 dB(A) SPL), the context tones of high saliency clearly increased the overall loudness (approximately 7 dB(A) SPL).

To make sure the context of low saliency was audible, and contexts of different saliency levels were distinguishable, two auditory tests were conducted with 16 new participants (13 females). Note that these tests were conducted in retrospect (i.e., after the experiments were finished). These participants were students and employees of the University of Passau, ranging in age from 19 to 40 years (*M* = 23.88, *SD* = 6.06). In the first auditory test, participants listened to a random sequence of trials consisting of either sound pairs without context or sound pairs combined with the low-saliency context. They were required to categorize the trials by an appropriate keypress (key *H* for sound pair without context, key *J* for sound pair with context). The one-sample *t* test of the sensitivity parameter *d′* (*M* = 2.20) revealed that it was significantly different from zero, *t*(15) = 7.52, *p* < .001, which means that the participants could easily detect the context of low saliency. In the second auditory test, participants listened to a random sequence of trials consisting of sound pairs with context of all three saliency levels and were asked to categorize them via keypress (key *H* for low saliency, key *J* for moderate saliency, and key *K* for high saliency). When calculating the hit and false-alarm rate for the comparison between the context of low and moderate saliency, the incorrect responses of categorizing the context of low or moderate saliency as being highly salient were excluded (around 4% of the trials for the former, around 18% of the trials for the latter). Similarly, in the comparison between the context of moderate saliency and high saliency, the incorrect responses of categorizing the moderately or highly salient context as being low salient were excluded (around 16% of the trials for the former, around 1% of the trials for the latter). The one-sample *t* test showed that both *d′* parameters (between low and moderate saliency, *M* = 1.24; between moderate and high saliency, *M* = 2.42) were significantly different from zero, *t*s > 7.81, *p*s < .001. Thus, participants could easily distinguish the contexts of different saliency levels.

Each trial comprised a prime presentation and a probe presentation. To create ignored repetition trials, three out of the four sounds were selected as target and distractor in the prime and probe presentations, with the restriction that the prime distractor was identical to the probe target (see Table 1). The parallel control trial for each ignored repetition trial was constructed by replacing the prime distractor with the remaining fourth sound. To prevent participants from anticipating response changes between prime and probe, we added attended repetition trials and their control counterparts. In attended repetition trials, three out of the four sounds were selected as target and distractor, with the restriction that the prime target was identical to the probe target. The parallel control trials were constructed by replacing the prime target with the remaining fourth sound. Since no hypothesis was made for attended repetition trials and their control counterparts, results of them are not reported here.

The basic set of experimental trials contained 48 trials, with 12 trials for each of the four trial types described above.^2^ The basic set was implemented four times: (1) with a 300-Hz context tone in both prime and probe presentations; (2) with a 700-Hz context tone in both prime and probe presentations; (3) with a 300-Hz context tone in the prime presentation and a 700-Hz context tone in the probe presentation; (4) with a 700-Hz context tone in the prime presentation and a 300-Hz context tone in the probe presentation. Note that Combinations 1 and 2 will be referred to as “context-repeated trials,” whereas Combinations 3 and 4 will be referred to as “context-changed trials.” This 192-trial set was repeated three times as there were three different saliency conditions, resulting in 576 trials in total. These 576 trials were presented in a random sequence in the experiment. For each trial, it was randomly decided on which side the prime target would be presented; the probe target would always be presented on the other side.

#### Procedure

Participants were familiarized with the experimental sounds and introduced to the task, followed by three training sessions. In the first training, presentations consisted of target and distractor pairs without context tones. Participants had to identify the target sound via key press. Participants had to achieve an accuracy of at least 60% in the preceding 15 training trials to pass the training. If the criterion was missed after 60 trials, participants were offered to quit or to repeat the training. In the second training, sound pairs were presented together with context tones. Participants were instructed that the context tones were task irrelevant and they should focus on the task itself. The criterion of the second training was identical to that of the first one. In the final training, participants responded to six prime–probe sound pair presentations. The timing of these final training trials was identical to the timing of the experimental trials.

An experimental trial started with a 20-ms metronome click, indicating the to-be-attended side. The prime presentation followed the click after a 500-ms cue–target interval. After the prime response, a 500-ms prime–probe interval elapsed, after which, the probe cue was presented on the opposite side of the prime cue. Following the cue–target interval, the probe sound pair was presented. Audio-visual feedback about the correctness of the prime and probe responses was given after each trial, followed by a 1,200-ms intertrial interval. Responses faster than 100 ms and slower than 3,000 ms were excluded from the analysis, and participants got warning messages.

The whole experiment comprised 24 blocks with 24 experimental trials in each block. After each block, feedback regarding error rate was presented. Participants were offered rest, and they could start the next block at their own discretion by pressing one of the response keys. The testing lasted for 75–90 minutes.

#### Design and analysis

The experiment comprised a 2 × 2 × 3 within-subjects design, with trial type (ignored repetition vs. control), context relation (repeated vs. changed), and context saliency (low vs. moderate vs. high) as independent variables. Apart from averaged reaction times and probe error rates, the probe response frequencies were analyzed.

**Fig. 1:**
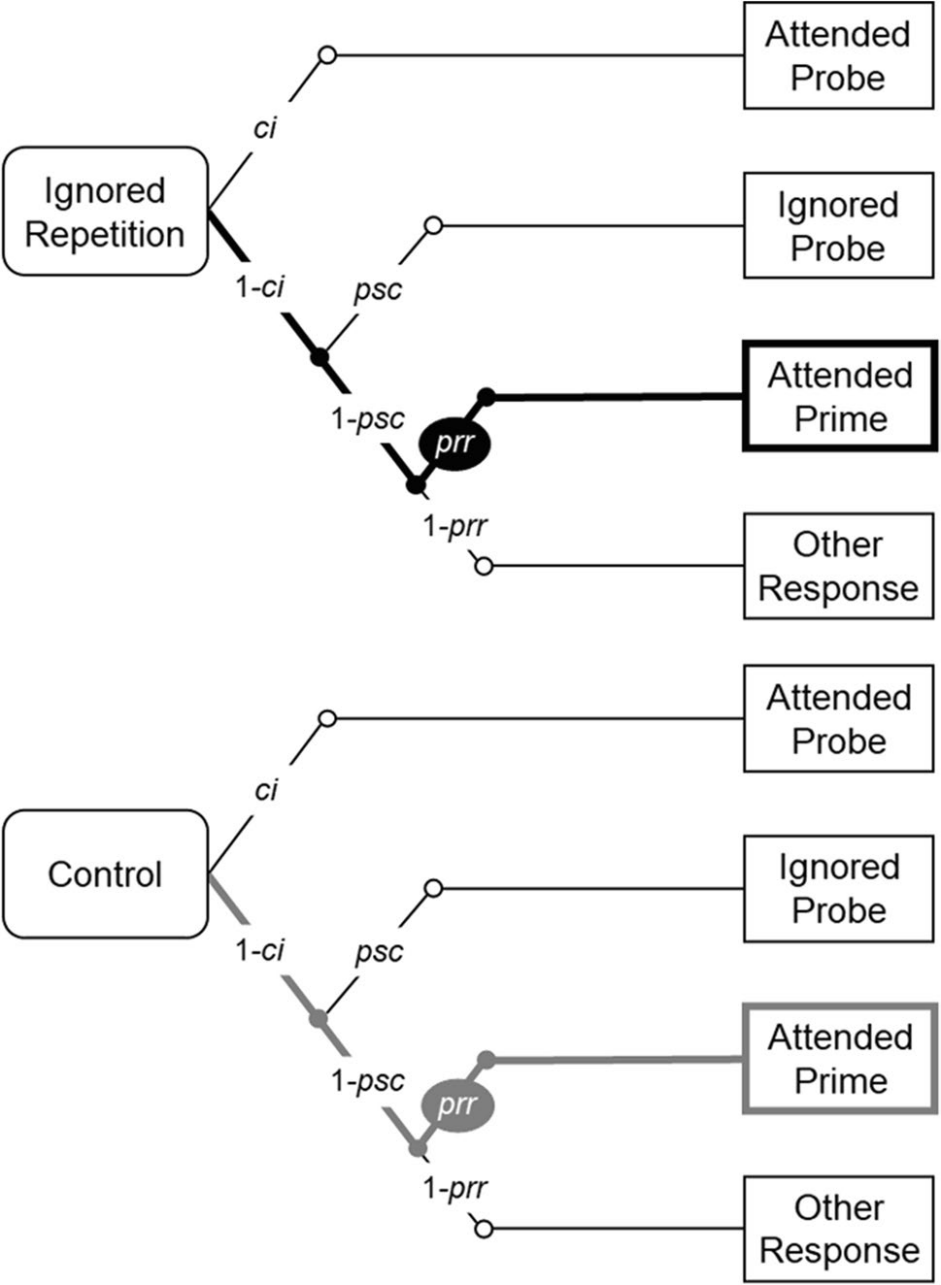
The baseline multinomial processing tree model for analyzing the probe response in ignored repetition and control trials

The multinomial processing tree (MPT) model introduced by Mayr and Buchner (2006) was used to estimate and compare the probability of the prime-response retrieval process for the different experimental conditions (see Hu & Batchelder, 1994, for a general introduction to multinomial processing tree modeling). This so-called baseline model (see Fig. 1) describes the occurrence of probe responses in the four-alternative identification task as a result of different processes. Correct identification of the probe target (with probability *ci*^3^) leads to a correct probe response. With probability 1 − *ci*, an erroneous response will occur, either for the probe distractor (with conditional probability *psc*) or, alternatively, with the former prime response key (with conditional probability *prr*). Finally, if prime-response retrieval does not take place (with conditional probability 1 − *prr*) an erroneous response with the remaining fourth response option is given.

The multinomial model allows for probability estimates and hypothesis testing. The stimulus–response binding and retrieval account predicts that the probability of prime response retrieval (i.e., *prr*) is larger when a stimulus is repeated than when it is changed. Accordingly, the probability of the *prr* parameter should be larger in ignored repetition trials (*prr*_IR_) than in control trials (*prr*_C_). This prediction was tested for each of the 2 × 3 (Context Relation × Context Saliency) conditions by calculating the goodness-of-fit of a model with the restriction of equal *prr* parameters between the ignored repetition and control conditions (i.e., *prr*_IR_ = *prr*_C_). A significant misfit of this restricted model to the empirical data will be evidence for the occurrence of the prime-response retrieval mechanism induced by the repetition of the prime distractor stimulus.

**Fig. 2:**
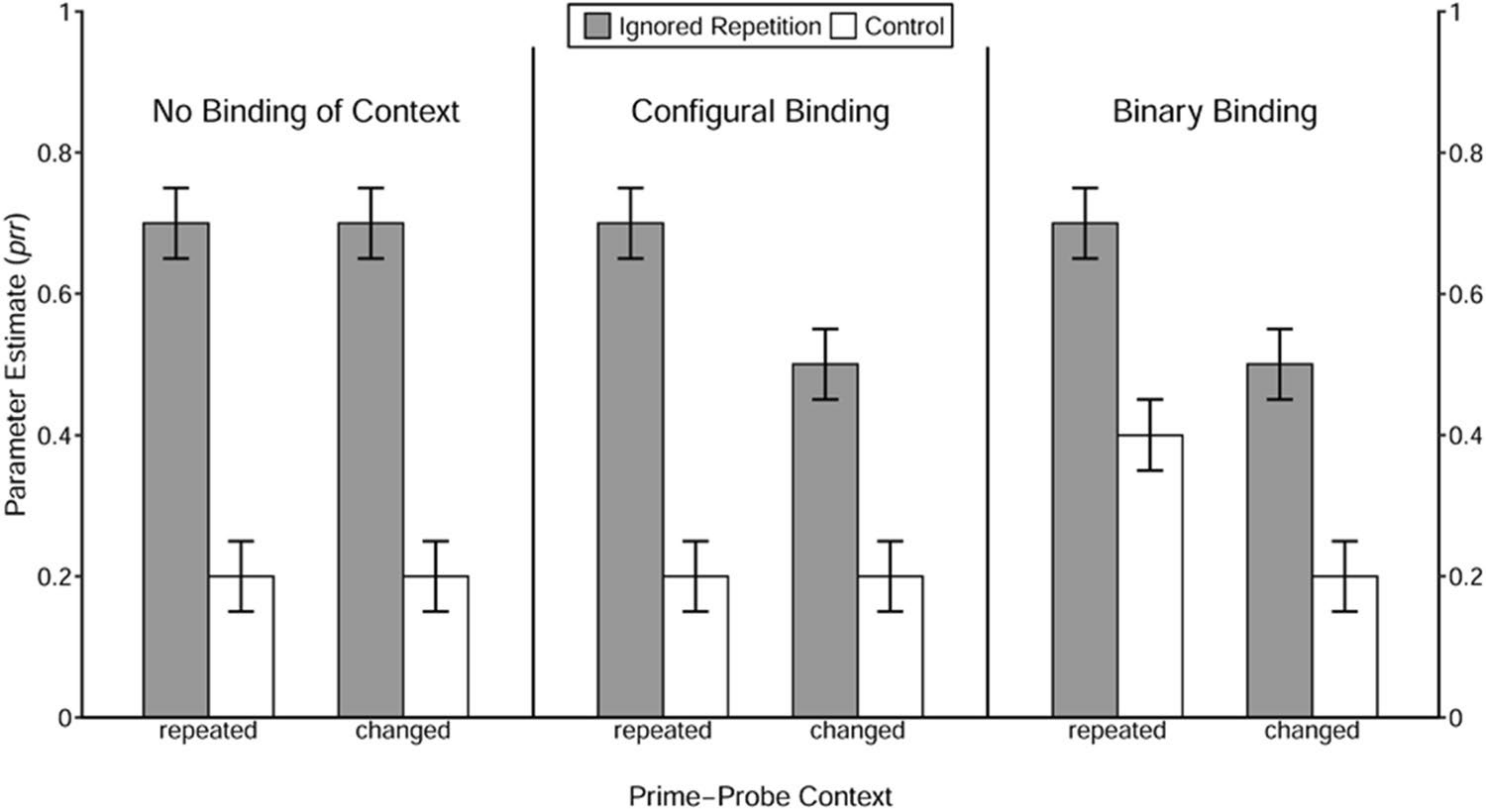
Example of prototypical result patterns for each type of context integration. *Note.* The *prr* parameter represents the retrieval of the prime response induced by the repetition of stimuli (the distractor and/or the context). The pattern on the left depicts the situation when retrieval of the prime response is not influenced by repetition of the context per se nor by repetition of the distractor and context combination, indicating that the context is not integrated into a stimulus–response episode. The pattern in the middle depicts the situation when the repetition of the context per se does not improve the retrieval of the prime response, but boosts distractor-induced prime-response retrieval, indicating that the context is involved in a configural binding with the prime distractor stimulus and the response. The pattern on the right depicts the situation when the repetition of the context per se improves the retrieval of the prime response, but does not facilitate distractor-induced prime-response retrieval, indicating that the context is involved in a binary binding with the response

Moreover, we tested whether the context was integrated into stimulus–response episodes and how context saliency influenced the type of context integration (see Fig. 2 for prototypical result patterns of each type of context integration). This was done in two steps: First, we tested for the presence of a binary binding between context and response and, second, for the presence of a configural binding among context, distractor stimulus, and response. A binary binding between context and response would be indicated by a significant prime-response retrieval effect induced by the repetition of the context per se. Therefore, for each of the three saliency conditions, the processing trees of the context-repeated and the context-changed conditions were integrated into one model (i.e., the joint model) and the goodness-of-fit of this joint model with the restriction of equal *prr*_C_ parameters between the context-repeated and the context-changed conditions was tested.

Next, the presence of a configural binding among context, distractor stimulus, and response was analyzed for each level of context saliency. Evidence for a configural binding is demonstrated if the prime-response retrieval effect induced by the repetition of the prime distractor stimulus is larger in the context-repeated than in the context-changed condition. This corresponds to an interaction effect between the factors context relation and trial type. The interaction analysis in MPT modeling requires reparameterization of the joint model (see Knapp & Batchelder, 2004, for details of reparameterization methods of MPT models, and please see the Appendix for detailed description of the reparameterized model and the interaction analysis used in the current study). In the reparametrized model, the prime-response retrieval effect induced by the repetition of the prime distractor stimulus can be represented by the difference between *prr*_IR_ and *prr*_C_ parameters (i.e., *prr*_IR_ − *prr*_C_). All MPT analyses were run with the multiTree software (Moshagen, 2010).

With respect to statistical power considerations, the contextual modulation of the prime-response retrieval effect was of central interest. The difference in the size of the prime-response retrieval effect induced by the repetition of the prime distractor stimulus between context-repeated and context-changed trials found in Mayr et al. (2018) was relatively small (ω = .03). To detect the contextual modulation of a similar size within each context-saliency condition, given desired levels of α = .05 and 1 − β = .80, approximately 8,721 trials in total were required for the model analysis. Since each participant maximally contributed 96 trials, that is, 24 trials for each 2 × 2 (Trial Type × Context Relation) condition, data had to be collected from 91 participants. We were able to collect usable data from 106 participants (i.e., 10,176 trials); thus, the power was slightly larger than what we had planned for (1 − β = .86). Note that in Experiment 1, 2A and 2B, *p* values for multiple comparisons were reported after Bonferroni-Holm correction (Holm, 1979). All sample size calculations were conducted using the G*Power program (Faul et al., 2009).

### Results

#### Analyses of reaction times and overall error rates

**Fig. 3:**
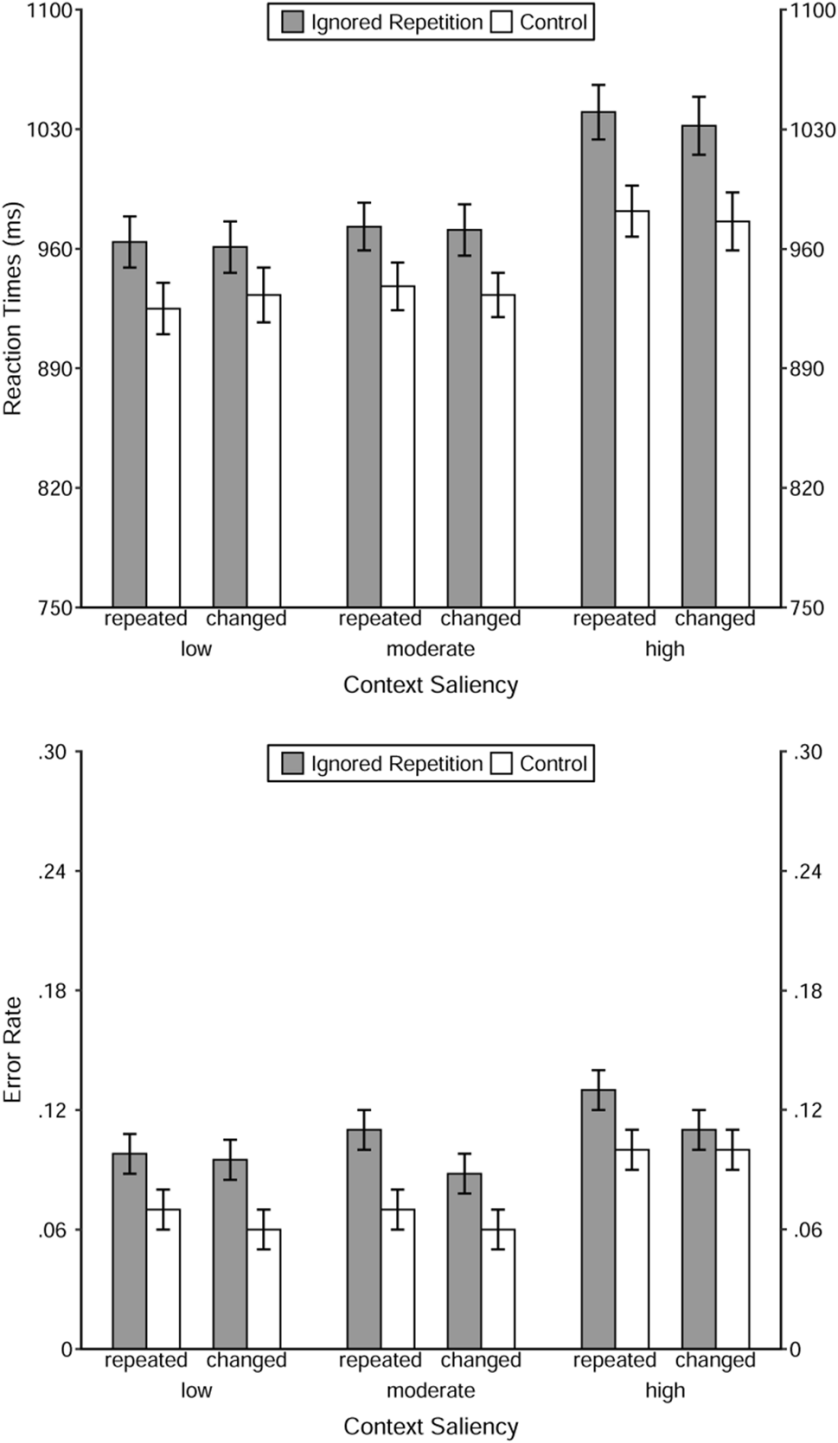
Reaction times (upper panel) and error rate (lower panel) as function of trial type, context relation, and context saliency in Experiment 1. *Note.* The error bars depict the standard errors of the means

A 2 (trial type: ignored repetition vs. control) × 2 (context relation: repeated vs. changed) × 3 (context saliency: low vs. moderate vs. high) repeated-measures multivariate analysis of variance (MANOVA) was applied to reaction times (see Table 2 for the main statistical results as well as Fig. 3 for an overview of the descriptive findings). The statistical analysis revealed a significant main effect of trial type, *F*(1, 105) = 62.60, *p* < .001, η_p_^2^ = .37. Probe responses were slower in the ignored repetition (*M*_RT_ = 990 ms) than in the control condition (*M*_RT_ = 948 ms), showing a significant negative priming effect in reaction times. There was also a significant main effect of context saliency, *F*(2, 104) = 42.20, *p* < .001, η_p_^2^ = .45. Probe responses were slower when context saliency was high (*M*_RT_ = 1,008 ms) than when it was moderate (*M*_RT_ = 953 ms), *F*(1, 105) = 85.21, *p* < .001, η_p_^2^ = .45, and low (*M*_RT_ = 946 ms), *F*(1, 105) = 28.55, *p* < .001, η_p_^2^ = .21. The difference of reaction times between the moderate-saliency and low-saliency conditions was not significant, *F*(1, 105) = 0.69, *p* > .99, η_p_^2^ = .01. Potentially, these results indicate that it was more difficult to identify or to focus on the task-relevant stimuli when the context was of high saliency. None of the other main or interaction effects was significant, all *F*s < 2.46, *p*s > .09.

The same MANOVA on error rates revealed a significant main effect of trial type, *F*(1, 105) = 39.91, *p* < .001, η_p_^2^ = .28, with higher error rates in the ignored repetition (*M*_error rate_ = .10) than in the control condition (*M*_error rate_ = .07). In other words, there was a significant negative priming effect in error rates. The main effect of context relation was also significant, *F*(1, 105) = 12.53, *p* < .01, η_p_^2^ = .11, showing that repetition of context in the probe presentation increased the probe error rates (for the context-repeated condition *M*_error rate_ = .10; for the context-changed condition, *M*_error rate_ = .08). Furthermore, there was a significant main effect of context saliency, *F*(2, 104) = 11.73, *p* < .001, η_p_^2^ = .18. The results pattern resembles that of the reaction times, specifically, the error rates were higher when context saliency was high (*M*_error rate_ = .11) than when it was moderate (*M*_error rate_ = .08), *F*(1, 105) = 23.40, *p* < .001, η_p_^2^ = .18, and when it was low (*M*_error rate_ = .08), *F*(1, 105) = 11.23, *p* < .01, η_p_^2^ = .10; whereas the difference in error rates between the moderate-saliency and low-saliency conditions was not significant, *F*(1, 105) = 0.13, *p* > .99, η_p_^2^ < .01. This pattern of results suggests that it might be more difficult to identify or to focus on the task-relevant stimuli when the context saliency was high. None of the interaction effects was significant, all *F*s < 2.52, *p*s > .11.

#### Multinomial analysis of categorial response frequencies

**Fig. 4:**
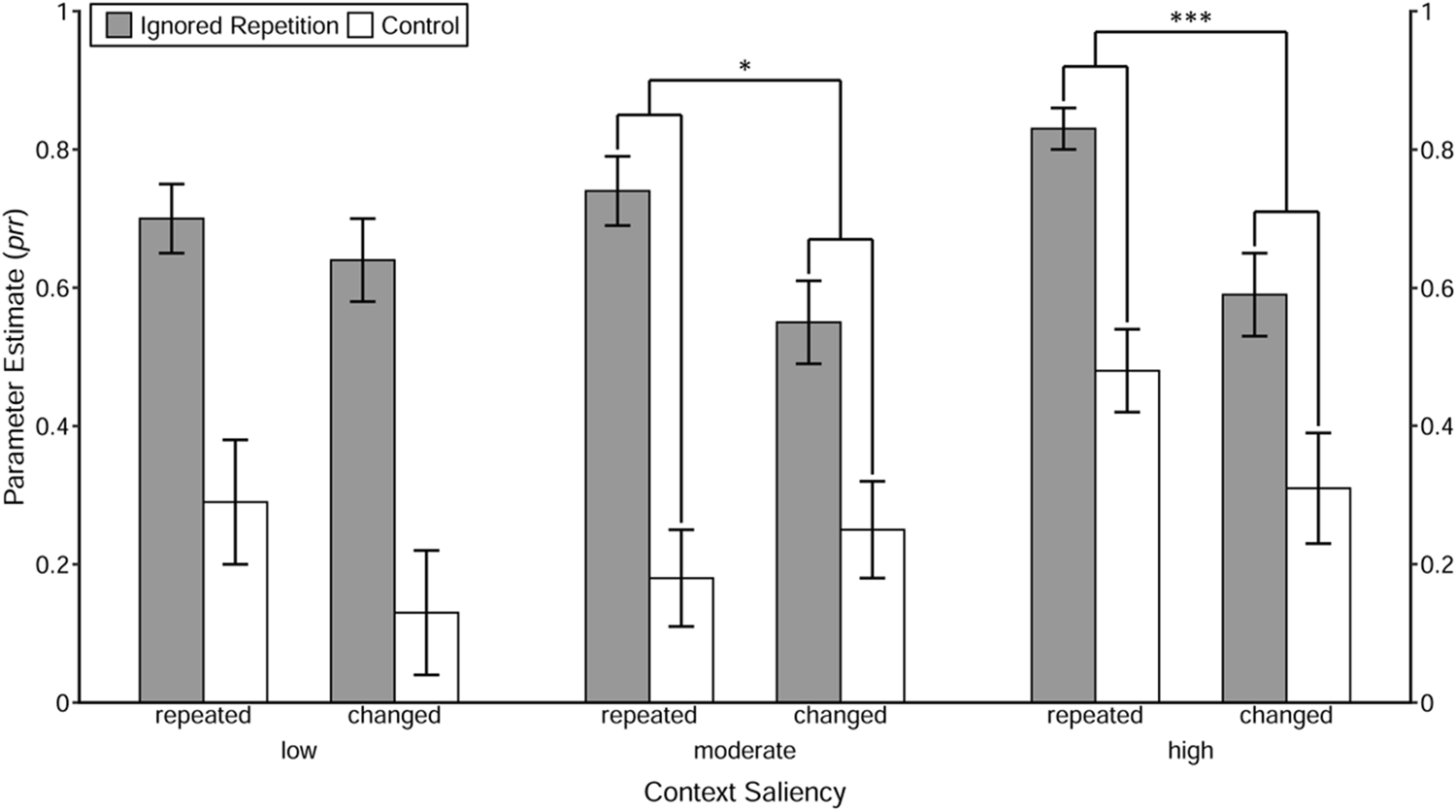
Probability estimates for the model parameters representing the probability of prime-response retrieval (*prr*) as a function of trial type, context relation, and context saliency in Experiment 1. *Note.* The error bars depict the standard errors of the means. Annotation shows significant comparisons indicating configural binding of the context. The symbols “*” and “***” indicate *p* < .05 and *p* < .001, respectively

The estimated prime-response retrieval parameters *prr*_IR_ and *prr*_C_ for all conditions are depicted in Fig. 4. Statistical results are summarized in Table 3. The goodness-of-fit tests of the baseline model with the restriction *prr*_IR_ = *prr*_C_ for each of the 2 × 3 (Context Relation × Context Saliency) conditions revealed that the restricted model had to be rejected for each context-relation condition, regardless of the saliency level, *G*^2^s > 6.83, *p*s < .01, ωs > .03. These results demonstrate clear evidence that the repetition of the prime distractor stimulus induced the retrieval of the prime response, which indicates that a binary binding was formed between the prime distractor stimulus and the response.

To investigate whether the repetition of context per se induced retrieval of the prime response (indicating evidence for binary binding between the context and the prime response), the *prr*_C_ parameters were then compared between the context-repeated and context-changed conditions when the saliency was low, moderate, and high, respectively. With the restriction of equivalence of the *prr*_C_ parameters between the context-repeated and context-changed conditions, the model fit the data in the low-saliency condition, *G*^2^(1) = 1.38, *p* = .24, ω = .01, and in the moderate-saliency condition, *G*^2^(1) = 0.50, *p* = .48, ω = .01. In the high-saliency condition, however, the misfit approached marginal significance, *G*^2^(1) = 2.56, *p* = .11, ω = .02. These results indicate that there was no evidence for binary binding between the context and the prime response when context saliency was low or moderate. When context saliency was high, there was a tendency of binary binding formation.

We then tested whether the retrieval of the prime response induced by the repetition of the prime distractor stimulus was larger for context-repeated than for context-changed trials (indicating evidence for configural binding among context, distractor, and response) under each context-saliency condition. In the interaction analysis, the abovementioned reparametrized model was used (see Appendix). With the restriction of equivalence of the prime-response retrieval effect induced by the repetition of the prime distractor stimulus (i.e., *prr*_IR_ − *prr*_C_) between context-repeated and context-changed trials, the goodness-of-fit tests showed a significant misfit in the moderate-saliency condition, *G*^2^(1) = 5.63, *p* = .02, ω = .02, and in the high-saliency conditions, *G*^2^(1) = 12.56, *p* < .001, ω = .04, but not in the low-saliency condition, *G*^2^(1) = 0.67, *p* = .41, ω = .01. Together, the results indicated that the context of moderate or high saliency was involved in a configural binding with the prime distractor stimulus and the prime response, whereas the context of low saliency was not.

### Discussion

The results showed that the saliency of the context is a crucial determinant in stimulus–response binding. Although a low saliency context was easily perceived (as the additional auditory test revealed), the repetition of this context per se neither led to an increase in prime response errors nor to a larger prime-response retrieval effect induced by the repetition of the prime distractor stimulus. However, the repetition of a moderately salient context significantly increased the prime-response retrieval effect induced by the repetition of the prime distractor stimulus, as compared with the condition without context repetition; but the moderate-saliency context itself did not lead to an increase of errors with the former prime response. As for the high-saliency condition, there was a tendency of a prime-response retrieval effect induced by the repetition of context information alone; and similar to the moderate-saliency condition, the context repetition significantly boosted the commission of prime-response errors due to the repetition of the prime distractor stimulus.

Together, the pattern of results indicates that saliency modulates the integration of context in a stimulus–response episode. Specifically, the results suggest that the context of low saliency was not integrated at all, and that the context of moderate saliency was involved in a configural binding. The context of high saliency, however, tended to be directly bound with the response. The fact that we only found a tendency of binary binding in the high-saliency condition may be due to insufficient context saliency. Possibly, the context was not loud enough to reach a sufficiently high-saliency level to enter into a binary binding. We did not want to exceed 80 dB(A) SPL for the overall sound compound due to ethical reasons. In order to conceptually replicate Experiment 1, saliency was manipulated differently in Experiment 2A and 2B—namely, by changing the value of the information carried by the context.

## Experiment 2A

It has been consistently found that stimuli carrying emotional (especially negative or unpleasant) information are more salient than those containing neutral or nonemotional information (e.g., Biggs et al., 2012; Niu et al., 2012; Ogawa & Suzuki, 2004). Therefore, Experiments 2A and 2B employed spoken vowels with either no (i.e., neutral) or a negative emotional pronunciation. Context sounds were as loud as the sound pair to keep the loudness-driven saliency constant between conditions. Given the comparable loudness, sounds without emotional pronunciation were considered to be as salient as the sound pair, comparable with the moderate saliency condition in Experiment 1. Therefore, the neutral context sounds were categorized as of moderate saliency. Due to their emotional information, the negative context sounds were considered more salient than the sound pair—thus, they were categorized as of high saliency. We expected that the context of high saliency should be bound directly with the response (i.e., binary binding), whereas the context of moderate saliency should be involved in a configural binding, as found for the moderate saliency condition in Experiment 1.

### Method

#### Participants

One hundred and fifty English-speaking participants (71 females) were recruited for the current experiment using Prolific (https://www.prolific.co) for online data collection. None of them reported suffering from any kind of hearing problems. Data sets of seven participants had to be rejected because of excessive error frequencies (>.50) in ignored repetition and control conditions (as compared with the average of .18), which suggested either inability to perform the task or unwillingness to follow the instructions. Data from the remaining 143 participants entered the analysis. Their age ranged from 18 to 47 years (*M* = 29, *SD* = 7.04). Participants received 3.30 pounds for their participation.

#### Materials, task, and procedure

Materials, task and procedure were identical to those in Experiment 1 with the following exceptions. Four context sounds (i.e., the vowel “a” pronounced in an angry way as well as the vowel “e” pronounced in a disgusted manner and their neutral counterparts) were recorded from a female speaker using an iPhone 8 cellphone. The sounds were cut to a length of 300 ms and set to the same loudness. We also ran an auditory test to make sure the emotional and neutral context sounds were distinguishable. Participants who took part in the auditory test for Experiment 1 participated in this test, too. They listened to a random sequence of trials consisting of sound pairs with either the emotional context or the neutral context and were required to categorize the contexts as being emotional or neutral by pressing an appropriate key (key *F* for neutral, key *J* for emotional). The one-sample *t* test showed that the *d′* parameter (*M* = 3.20) was significantly different from zero, *t*(15) = 4.94, *p* < .001. Therefore, participants could easily distinguish between the emotional and neutral context sounds.

The four stimulus sounds were assigned to four basic keyboard keys, with “frog,” “piano,” “drum,” and “bell” assigned to keys *F*, *V*, *J,* and *N*, respectively. Participants were instructed to respond to the frog and the piano sounds using their middle and index fingers of the left hands, and to respond to the drum and the bell sounds using their middle and index fingers of the right hands.

To shorten the experiment for online data collection, the original 48 trials in the basic set were reduced to 32 trials, with the restriction that stimuli occurred equally often. The basic set was repeated four times (two times in the context-repeated condition and two times in the context-changed condition), resulting in a set of 128 trials. These 128 trials were duplicated (once for each of the two saliency conditions), thus there were 256 trials in total, which were presented in a random sequence.

The experiment was programmed using PsychoPy3 (Peirce et al., 2019), and was hosted on the Pavlovia platform (https://pavlovia.org). Participants from Prolific received an invitation to the experiment and were linked to Pavlovia. Participants were first instructed to use a headphone and to adjust the loudness to a comfortable level. After being introduced to the task, participants were familiarized with the four stimulus sounds. The training sessions were similar to those in Experiment 1, but the criterion to pass each training was set to 42% correct in 12 trials to reduce the overall task duration. Timing of the experimental trial was identical to Experiment 1, with the exception that the intertrial interval was prolonged to 2,000 ms. The experiment comprised 16 blocks with 16 experimental trials in each, and it took 30 to 45 minutes to finish.

#### Design and analysis

Experiment 2A comprised a 2 × 2 × 2 within-subjects design, with trial type (ignored repetition vs. control), context relation (repeated vs. changed), and context saliency (moderate vs. high) as independent variables. Dependent variables were averaged reaction times, overall probe error rates, and, most importantly, probe response frequencies. The analysis of the categorical response frequencies followed the same rationale as in Experiment 1.

The current experiment contained fewer trials in each of the 2 × 2 × 2 (Trial Type × Context Relation × Context Saliency) conditions as compared with Experiment 1 (i.e., 16 trials vs. 24 trials). Sample-size calculations followed the rationale of Experiment 1: To detect the contextual modulation of a similar effect size (i.e., ω = .03), given desired levels of α = .05 and 1 − β = .80, probe response data had to be collected from 136 participants. The final sample comprised 143 participants (i.e., 9,152 trials), so the power was slightly larger (.82) than originally planned for.

### Results

#### Analysis of reaction times and overall error rates

**Fig. 5:**
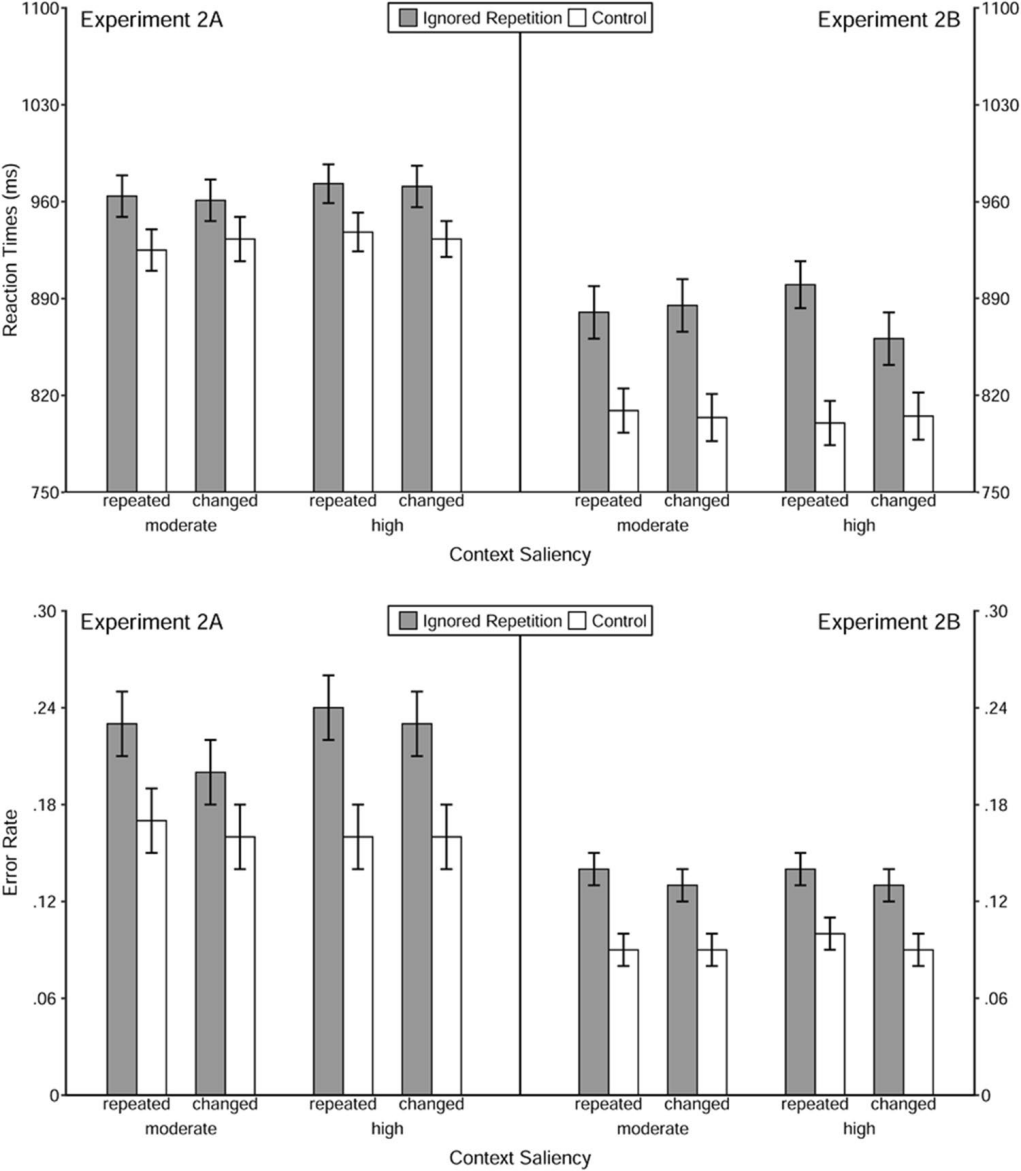
Reaction times (upper panel) and error rate (lower panel) as function of trial type, context relation, and context saliency in Experiment 2A and 2B. *Note.* The error bars depict the standard errors of the means

A 2 (trial type: ignored repetition vs. control) × 2 (context relation: repeated vs. changed) × 2 (context saliency: moderate vs. high) repeated-measures MANOVA was applied to reaction times (see Table 4 for the main statistical results as well as Fig. 5 for an overview of the descriptive finding). The main effect of trial type was significant, *F*(1, 142) = 84.85, *p* < .001, η_p_^2^ = .37; the probe responses were slower in ignored repetition trials (*M*_RT_ = 963 ms) than in control trials (*M*_RT_ = 878 ms), revealing a significant negative priming effect in reaction times. However, neither context relation nor context saliency affected reaction times—for the former, *F*(1, 142) = 0.96, *p* = .33, η_p_^2^ = .01, for the latter, *F*(1, 142) = 0.62, *p* = .43, η_p_^2^ < .01. None of the interaction effects was significant, all *F*s < 0.35, *p*s > .55.

The same MANOVA on error rates revealed a significant main effect of trial type as well, *F*(1, 142) = 70.34, *p* < .001, η_p_^2^ = .33; probe responding in ignored repetition trials (*M*_error rate_ = .23) comprised more errors than that in control trials (*M*_error rate_ = .16), showing a negative priming effect in error rates. The main effect of context relation was significant, *F*(1, 142) = 5.46, *p* = .02, η_p_^2^ = .04, with a higher error rate when context was repeated (*M*_error rate_ = .20) than when it was changed (*M*_error rate_ = .19), which replicates the findings in Experiment 1. The main effect of context saliency was not significant, *F*(1, 142) = 1.20, *p* = .28, η_p_^2^ = .01. None of the interaction effects reached the significance level, either—all *F*s < 2.92, *p*s > .08.

#### Multinomial analysis of categorical response frequencies

**Fig. 6:**
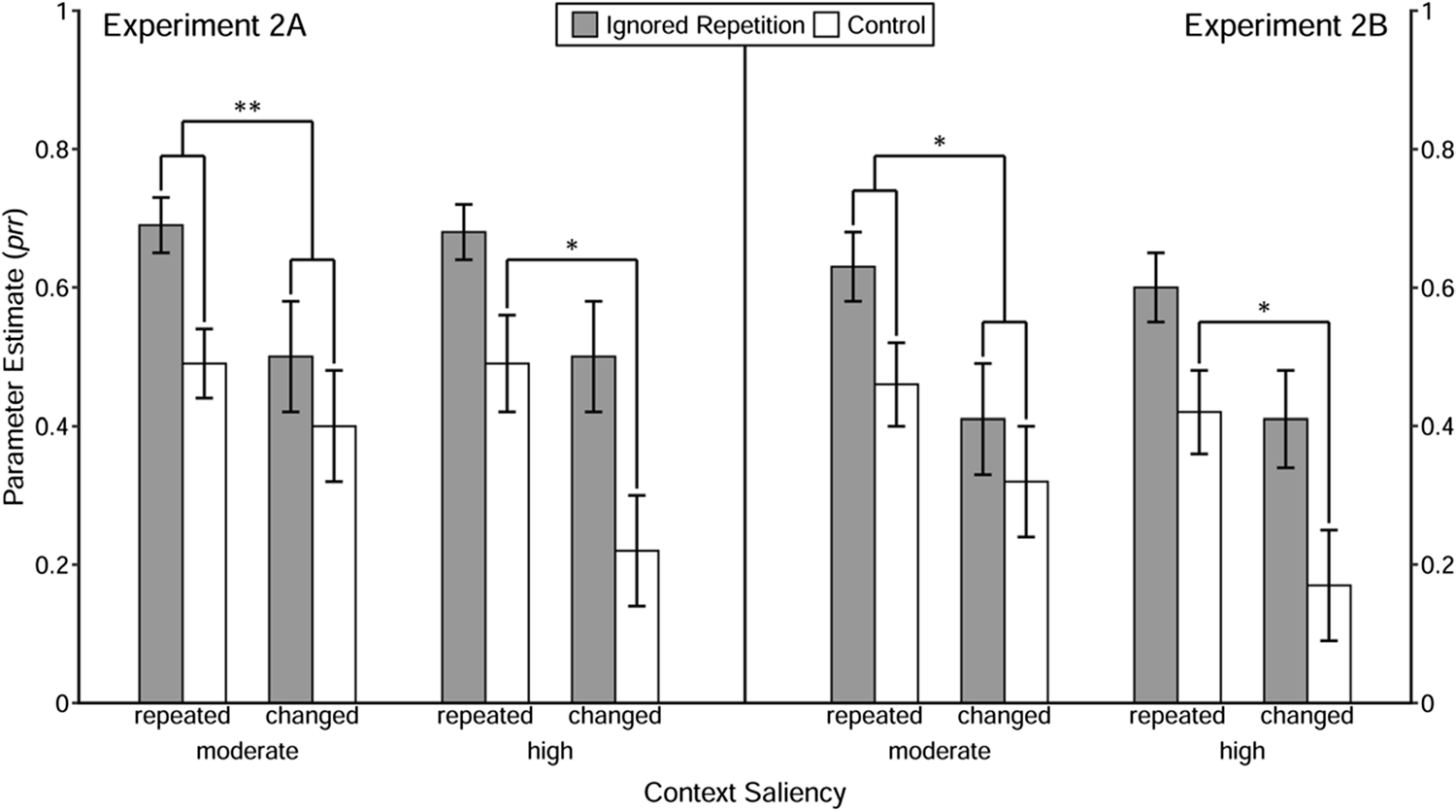
Probability estimates for the model parameters representing the probability of prime-response retrieval (*prr*) as a function of trial type, context relation, and context saliency in Experiment 2A and 2B. *Note.* The error bars depict the standard errors of the means. Annotation shows significant comparisons indicating configural and binary binding of the context. The symbols “*” and “**” indicates *p* < .05 and *p* < .01, respectively

Estimated *prr* parameters are depicted in Fig. 6. Statistical results are summarized in Table 5. First, it was tested whether the repetition of the prime distractor stimulus induced the retrieval of the prime response, suggesting that a binary binding between prime distractor and response had been formed. Results of the goodness-of-fit tests of the baseline model with the restriction *prr*_IR_ = *prr*_C_ showed evidence for the binary binding between prime distractor and response when context saliency was high, no matter whether the context was repeated, *G*^2^(1) = 4.40, *p* = .04, ω = .03, or changed, *G*^2^(1) = 6.25, *p* = .01, ω = .04. However, when context saliency was moderate, increased retrieval of the prime response due to repetition of the prime distractor stimulus was only found in the context-repeated condition, *G*^2^(1) = 4.53, *p* = .03, ω = .03, but not in the context-changed condition, *G*^2^(1) = 0.77, *p* = .38, ω = .01.

Then, to investigate whether it is solely the repetition of context that induced retrieval of the prime response (indicating evidence of binary binding between the context and the prime response), a restricted model with equivalent *prr*_C_ parameters in the context-repeated and the context-changed conditions was tested. Results revealed a significant misfit of the restricted model in the high-saliency condition, *G*^2^(1) = 5.86, *p* = .02, ω = .03, but not in the moderate-saliency condition, *G*^2^(1) = 0.64, *p* = .42, ω = .01. This suggests that the context of high saliency was involved in a binary binding with the response, whereas the context of moderate saliency was not.

Finally, a reparametrized model was used to test the configural binding hypothesis. With the restriction of equivalence of the prime-response retrieval effect (i.e., *prr*_IR_ − *prr*_C_) between context-repeated and context-changed trials, the goodness-of-fit tests showed a significant misfit in the moderate-saliency condition, *G*^2^(1) = 7.82, *p* < .01, ω = .03, but not in the high-saliency condition, *G*^2^(1) = 0.37, *p* = .54, ω = .01. These results indicate that the context of moderate saliency was involved in a configural binding with the prime distractor stimulus and the prime response, whereas the context of high saliency was not.

### Discussion

Experiment 2A demonstrates that the repetition of a highly salient context per se significantly increases the probability of retrieving the prime response as compared with a condition without context repetition. In contrast, the repetition of a context of moderate saliency did not retrieve the prime response on its own but increased the prime-response retrieval effect induced by the repetition of the prime distractor stimulus as compared with a changed context.

The results of Experiment 2A replicate the findings from Experiment 1, again revealing evidence of configural binding among the moderately salient context, the prime distractor, and the response. Furthermore, the results provide evidence for a binary binding between the highly salient context and the response (for which only a tendency was found in Experiment 1). In sum, results from Experiment 2A underline the conclusion from Experiment 1 that the specific binding between context and other elements of an episode is determined by context saliency. Specifically, a context of high saliency is involved in a binary binding with the response, whereas a context of moderate saliency is involved in a configural binding among several elements (stimuli and response).

## Experiment 2B

### Method

#### Participants

Among the 150 German-speaking participants (66 females), 30 of whom were students of the University of Passau, the remaining participants were from the Prolific platform. Data sets of three participants had to be excluded because of exceeding error rates (>.5) in ignored repetition and control conditions (as compared with the average of around .11), which suggests either unwillingness or inability to follow the instruction. The remaining 147 participants whose data sets entered into the analysis ranged in age from 18 to 41 years (*M* = 26, *SD* = 5.62). Students from the University of Passau received course credit for their participation, whereas participants from the Prolific platform received 3.30 pounds monetary reward.

#### Materials, task, procedure, and design

Materials, task, procedure and design were identical to those in Experiment 2A. To detect the contextual modulation of a similar effect size as in Experiment 2A (i.e., ω = .03), given desired levels of α = .05 and 1 − β = .80, probe response data had to be collected from a sample of 136 participants. The final sample comprised 147 participants (i.e., 9,408 trials), so the power was slightly larger than what we had planned for (1 − β = .83).

### Results

#### Analysis of reaction times and overall error rates

A 2 (trial type: ignored repetition vs. control) × 2 (context relation: repeated vs. changed) × 2 (context saliency: moderate vs. high) repeated-measures MANOVA was applied to reaction times and error rates. The main effect of trial type was significant in reaction times, *F*(1, 146) = 115.65, *p* < .001, η_p_^2^ = .44, and in error rates, *F*(1, 146) = 52.85, *p* < .001, η_p_^2^ = .27. The probe responses were slower and more error prone in ignored repetition trials (*M*_RT_ = 883 ms, *M*_error rate_ = .13) than in control trials (*M*_RT_ = 805 ms, *M*_error rate_ = .09), revealing a negative priming effect in both dependent measures. The manipulation of context (i.e., context relation or context saliency) did not affect reaction times, whereas a marginally significant main effect of context relation was found in error rates, *F*(1, 146) = 3.88, *p* = .05, η_p_^2^ = .03, with a relatively higher error rate when the context was repeated (*M*_error rate_ = .12) than when it was changed (*M*_error rate_ = .11). None of the interaction effects was significant, all *F*s < 3.74, *p*s > .05.

#### Multinomial analysis of categorical response frequencies

Firstly, the prime-response retrieval effect induced by the repetition of the prime distractor stimulus was investigated. With the restriction *prr*_IR_ = *prr*_C_, the restricted model had to be rejected when the context saliency was high, regardless of whether the context was repeated, *G*^2^(1) = 4.49, *p* = .03, ω = .03, or changed, *G*^2^(1) = 5.03, *p* = .02, ω = .03. When context saliency was moderate, the restricted model had to be rejected only when the context was repeated, *G*^2^(1) = 5.29, *p* = .02, ω = .04, but not when the context was changed, *G*^2^(1) = 1.80, *p* = .18, ω = .02.

Then, the prime-response retrieval effect induced by the repetition of the context per se was investigated. To this end, a restricted model with equivalent *prr*_C_ parameters in the context-repeated and the context-changed conditions was tested. Results revealed a significant misfit of the restricted model in the high-saliency condition, *G*^2^(1) = 4.04, *p* = .04, ω = .02, but not in the moderate-saliency condition, *G*^2^(1) = 0.51, *p* = .47, ω = .01. This suggests that the context of high saliency was involved in a binary binding with the response, whereas the context of moderate saliency was not.

Finally, a reparametrized model was used to test the configural binding hypothesis. With the restriction of equivalence of the prime-response retrieval effect (i.e., *prr*_IR_ − *prr*_C_) between context-repeated and changed trials, the goodness-of-fit tests showed a significant misfit in the moderate-saliency condition, *G*^2^(1) = 4.66, *p* = .03, ω = .02, but not in the high-saliency condition, *G*^2^(1) = 0.18, *p* = .67, ω < .01. These results indicate that the context of moderate saliency was involved in a configural binding with the prime distractor stimulus and the prime response, whereas the context of high saliency was not.

### Discussion

With a different sample of participants, Experiment 2B showed the identical results pattern as in Experiment 2A. Specifically, the prime-response retrieval effect induced by the repetition of the context per se was significant in the high-saliency condition, but not in the moderate-saliency condition. However, the contextual modulation of the prime-response retrieval effect induced by the repetition of the prime distractor stimulus was significant in the moderate-saliency condition, but not in the high-saliency condition. Together, results in Experiment 2B show again, that the context of high saliency is involved in a binary binding with the response, whereas the context of moderate saliency is involved in a configural binding together with the prime distractor stimulus and the response.

## General discussion

The goal of the current study was to elucidate the integration of context in a stimulus–response episode, with a focus on the role of saliency. To this end, the saliency of an auditory context was manipulated by changing its loudness (Experiment 1) and emotional valence (Experiments 2A and 2B). Despite the different ways of the saliency manipulation, the results of all experiments showed a similar pattern of results in the moderate-saliency condition: the prime-response retrieval effect induced by the repetition of the prime distractor stimulus was larger when the context was repeated than when it was changed, but the context repetition alone did not retrieve the prime response. This constitutes a replication of the findings reported by Mayr et al. (2018). More importantly, in the high-saliency condition, results from Experiments 2A and 2B show that the repetition of the context did not increase the prime-response retrieval effect induced by the repetition of the prime distractor stimulus, but it retrieved the prime response on its own. Note that in the high-saliency condition of Experiment 1, results only revealed a tendency of such a direct response retrieval induced by context repetition, presumably due to insufficient context saliency. On the other hand, the repetition of the highly salient context in Experiment 1 boosted the prime-response retrieval effect induced by the repetition of the prime distractor. As for the low-saliency condition, results from Experiment 1 show that repetition of context per se did not retrieve the prime response, and that repetition of context did not boost the probability of retrieving the prime response induced by the repetition of the prime distractor stimulus, either. Taken together, Experiments 1, 2A, and 2B provide empirical evidence that saliency is a determinant of context integration. Specifically, context of low saliency is not integrated into a stimulus–response episode at all, context of moderate saliency is involved in a configural binding, whereas context of (sufficiently) high saliency enters into a binary binding with the response.

The integration of context as a function of saliency level is consistent with proposed assumptions about binding principles (Hommel, 2004). Following this notion, a binary binding between a task-irrelevant stimulus and a response is only formed when the stimulus is salient enough to pass a certain integration threshold. If this threshold is missed, the stimulus will not be integrated at all (e.g., Dutzi & Hommel, 2009). This pattern describes what we found in the (sufficiently) high-saliency versus low-saliency conditions in the current study. However, the findings of configural binding structures in Experiments 1, 2A, and 2B might extend this binding principle: If a stimulus passes the basic integration threshold (and is therefore bound), a second saliency threshold will then determine the specific binding structure (i.e., binary vs. configural). When the saliency of a stimulus is sufficient to be integrated but misses the threshold for binary binding, it will enter into a configural binding. Otherwise, it will be bound with the response in a binary fashion.

The distinction between binary and configural bindings based on the saliency level may result from the influence of saliency on the perception of a stimulus—that is, whether the stimulus is perceived as an individual object or not. Referring to the figure–ground segmentation literature, there is evidence that saliency determines whether a part of a stimulus is perceived as a *figural element/object* or the *background* of other parts (Hoffman & Singh, 1997; Wagemans et al., 2012). In essence, with other properties being equal, the more salient part will be assigned the status as the “figure” in a display. Transferring this finding into the auditory modality, it is likely that the auditory contextual stimulus of high saliency will be perceived as an individual object, whereas the stimulus of relatively lower saliency may be perceived as the background of the other stimuli. Furthermore, given that the latter is presumably more similar to the other stimuli (in the sense of saliency level operationally defined by loudness and emotional valence in the current study) than the former, the latter may be more likely to be perceptually grouped with the other stimuli (Wagemans et al., 2012), thereby forming a compound.^4^ Together, the “figure” object, which is presumably distinguishable from other stimuli, is more likely to enter a binary binding with the response (Moeller et al., 2016), whereas the “background” may be involved in a configural binding as a part of a compound. This notion fully conforms to what we found in the current study.

The current findings bear resemblance to findings in learning—namely, *configural* and *elemental* associations in classical conditioning (for a review, see Pearce & Bouton, 2001). While the former assumes an association between a compound of elements with a reinforcer (Shanks et al., 1998), the latter assumes unitary association between each element and the reinforcer (Rescorla & Wagner, 1971). Recently, these two types of associations were found to coexist but to be supported by different neural systems (for a review, see Honey et al., 2014). For example, Iordanova et al. (2009) found that healthy rats could form both elemental and configural association, but lesions in the hippocampus left rats reliant on elemental associations, which means the hippocampus is involved in configural but not in elemental associations. For another example, the retrosplenial cortex, which is involved in contextual fear conditioning, was found to contribute more to the configural approach (Todd et al., 2017). Assuming an overlap between the mechanisms involved in binding and conditioning, the distinction by the second saliency threshold that decides whether the context is involved in configural or binary binding might have a neural basis. With that being said, future studies are required to investigate the neural basis of our findings.

Note that the current study did not reveal significant contextual modulation of the negative priming effect in reaction times or in overall error rates. This is consistent with the previous study by Mayr et al. (2018), in which the prime-response retrieval process was found to be the only mechanism underlying the negative priming effect that was sensitive to contextual modulation. However, it is noteworthy that in the visual modality the contextual modulation of the negative priming effect has been consistently found (e.g., Chao, 2009; Chao & Yeh, 2008), reasons for this difference between modalities should be investigated in future studies.

To sum up, the current study manipulated the saliency property of context to investigate its influence on the integration of context in stimulus–response episodes. Results show that only contextual stimuli of sufficient saliency can be integrated into a stimulus–response episode, entering into either a configural or a binary structure, depending on the context saliency level. Taken together, the current study provides detailed insights into the architecture of bindings between completely task-irrelevant features and actions, and thus sheds light on how contextual information influences human behavior.

## Data Availability

The data and programming code for data analysis of all experiments are available at PsychArchives (10.23668/psycharchives.5340). None of the experiments was preregistered. This work is part of the doctoral dissertation by Ruyi Qiu.

## Appendix

Standard statistical testing procedures of MPT models only concern testing the equivalence between model parameters. To analyze interaction effects—in this case, whether the prime-response retrieval effect (as the difference between the *prr* parameters in the ignored repetition condition, represented by “IR,” and the control condition, represented by “C”) is larger in the context repeated than in the context changed condition—requires a so-called reparameterization (see Knapp & Batchelder, 2004). This process introduces new parameters to represent certain effects. With these new parameters, it can be tested whether certain effects (here: *prr*_IR_ − *prr*_C_) differ between conditions (here, the context-repeated vs. the context-changed condition). The interaction analysis used in the current study is described in the following.

The reparameterization is applied to the baseline model introduced by Mayr and Buchner (2006) for testing the prime-response retrieval effect (see Fig. 1 in the main text). Assuming a joint model of the two context-relation conditions (repeated vs. changed), the *prr* parameters in each baseline model can be presented with the subscript “REP” for the context-repeated condition, and the subscript “CH” for the context-changed condition, respectively.

Referring to Knapp and Batchelder (2004), there are two basic methods to reparametrize MPT models, called Method A and B, respectively. If we have two parameters of interest, θ_1_ and θ_2_ (with the order restriction 0 ≤ θ_1_ ≤ θ_2_ ≤ 1) in the original model, according to Method A, a new parameter α can be introduced to reparametrize the original model, with α = θ_1_ / θ_2_. As for Method B, instead, a new parameter β can be introduced to reparametrize the original model, with β = (θ_2_ − θ_1_) / (1 − θ_1_). Method A and B can be applied additively if necessary, as long as the resulting reparametrized model is statistically equivalent to the original model (i.e., results of the goodness-of-fit test of these models with the same set of empirical data should be identical) and the reparametrized model itself is identifiable. Both Method A and B are used for the current analysis. The procedure is described in detail in the following.

First, Method B is applied to the joint model to represent the prime-response retrieval effects (i.e., the difference between the *prr*_IR_ and the *prr*_C_ parameters) with the context repeated (i.e., *prr*_IR_REP −_ *prr*_C_REP_) or changed (i.e., *prr*_IR_CH −_ *prr*_C_CH_). The resulting model is called the *β-reparametrized model*. In the β-reparametrized model for each saliency condition, there are two β parameters, one for the context-repeated condition (β__REP_ = [*prr*_IR_REP −_ *prr*_C_REP_] / [1 − *prr*_C_REP_]) and one for the context-changed condition (β__CH_ = [*prr*_IR_CH −_ *prr*_C_CH_] / [1 − *prr*_C_CH_]). For empirical data from each saliency condition of the current experiments, the goodness-of-fit test of the β-reparametrized model yielded the same result as the one for the joint model, *G*^2^(0) = 0. The local identifiability test (repeatedly estimating 1,000 times) using multiTree (Moshagen, 2010) showed almost no deviations in any of the parameter estimates (all deviations ≤0.00001). Similarly, the simulated identifiability test yielded an average deviation smaller than 0.00001. In the β-reparametrized model, the prime-response retrieval effect in the context-repeated (i.e., *prr*_IR_REP −_ *prr*_C_REP_) and context-changed conditions (i.e., *prr*_IR_CH −_ *prr*_C_CH_) can be represented as follows:

1 $$prr\text{IR}\_\text{REP}- prr\mathrm{C}\_\text{REP}=\beta \_\text{REP}\cdot  \left(1- prr\mathrm{C}\_\text{REP}\right),$$

2 $$prr\text{IR}\_\text{CH}- prr\mathrm{C}\_\text{CH}=\beta \_\text{CH}\cdot  \left(1- prr\mathrm{C}\_\text{CH}\right).$$

To determine whether the prime-response retrieval effect in the context-repeated condition is different from that in the context-changed condition, we tested whether the quotient of (*prr*_IR_REP_ − *prr*_C_REP_) and (*prr*_IR_CH_ − *prr*_C_CH_) equals 1. Combining Eqs. 1 and 2, the following relation is tested:

3 $$\frac{\beta_{\_\text{REP}}}{\beta_{\_\text{CH}}}\bullet \frac{1-{prr}_{\mathrm{C}\_\text{REP}}}{1-{prr}_{\mathrm{C}\_\text{CH}}}=1.$$

If the *prr*_C_REP_ parameter is statistically equivalent to the *prr*_C_CH_ parameter in the joint model, a nested model with an additional restriction β__REP_ = β__CH_ can be built to test whether the β parameters are equal as well. Note that this nested model is equivalent to a model with the restriction (*prr*_IR_REP_ − *prr*_C_REP_) = (*prr*_IR_CH_ − *prr*_C_CH_). This nested model analysis was applied in the low-saliency, moderate-saliency, and high-saliency conditions in Experiment 1 as well as the moderate-saliency condition in Experiments 2A and 2B, because the restricted model with the restriction *prr*_C_REP_ = *prr*_C_CH_ did not yield significant misfit with the empirical data in these conditions.

When the model with the restriction *prr*_C_REP_ = *prr*_C_CH_ has to be rejected for the empirical data, which is the case in the high-saliency condition of Experiments 2A and 2B, the nested model mentioned above can no longer test whether Eq. 3 is satisfied or not. Thus, further reparameterization is required, which is described in the following.

An equal transformation of Eq. 3 will be:

4 $$\frac{\beta_{\_\text{CH}}}{\beta_{\_\text{REP}}}=\frac{1-{prr}_{\mathrm{C}\_\text{REP}}}{1-{prr}_{\mathrm{C}\_\text{CH}}}.$$

If Eq. 4 is satisfied, then Eq. 3 is satisfied as well, which means there is no statistical difference between (*prr*_IR_REP_ − *prr*_C_REP_) and (*prr*_IR_CH_ − *prr*_C_CH_). Instead, if Eq. 4 is not satisfied, then Eq. 3 will not be satisfied, indicating that (*prr*_IR_REP_ − *prr*_C_REP_) and (*prr*_IR_CH_ − *prr*_C_CH_) are statistically different from each other.

To test whether Eq. 4 is satisfied or not, quotients on the left and right side of Eq. 4 need to be represented by new parameters, because MPT model analysis does not allow for directly testing the equivalence of two quotients of model parameters, but allows for testing the equivalence of two model parameters. Here, Method A can be applied to the β-reparametrized model to represent these quotients with two new α parameters. Specifically, we can get an α_beta parameter which equals (β__CH_ / β__REP_), and an α_*prr*_C_ parameter which equals (1 − *prr*_C_REP_) / (1 − *prr*_C_CH_). Equation 4 can then be represented as:

5 $$\upalpha \_\text{beta}=\upalpha \_\text{prrC}.$$

Now, with these two new *α* parameters, we get a fully reparametrized model. This fully reparametrized model yielded the same model fit as the joint model for empirical data from the high-saliency condition in Experiment 2A, *G*^2^(0) = 0, and in Experiment 2B, *G*^2^(0) = 0. In addition, the local identifiability test using multiTree showed almost no deviations in any of the parameter estimates (all deviations ≤0.00005). Similarly, the simulated identifiability test yielded an average deviation smaller than 0.00001. With this fully reparametrized model, we can directly test the difference between (*prr*_IR_REP_ − *prr*_C_REP_) and (*prr*_IR_CH_ − *prr*_C_CH_) by restricting α_beta = α*_prr*_C_. Parameter estimates in the joint and reparametrized models are displayed in Appendix Tables 6, 7 and 8 for Experiment 1, 2A, and 2B, respectively.

**Table 6:** Parameter estimates from the joint and reparametrized models for Experiment 1

Context Repeated						Context Changed						New Parameters				
Ignored Repetition			Control			Ignored Repetition			Control							
*ci*	*psc*	*prr*	*ci*	*psc*	*prr*	*ci*	*psc*	*prr*	*ci*	*psc*	*prr*	β__CH_	β__REP_	α_beta	α*_ prr*_C_	*G*^*2*^
Joint Model (low-saliency condition)																
0.90 (0.01)	0.58 (0.03)	0.70 (0.05)	0.94 (0.01)	0.82 (0.03)	0.29 (0.09)	0.91 (0.01)	0.67 (0.03)	0.64 (0.06)	0.95 (0.01)	0.87 (0.03)	0.13 (0.09)					0
β-reparametrized Model (low-saliency condition)																
0.90 (0.01)	0.58 (0.03)		0.94 (0.01)	0.82 (0.03)	0.29 (0.09)	0.91 (0.01)	0.67 (0.03)		0.95 (0.01)	0.87 (0.03)	0.13 (0.09)	0.59 (0.08)	0.58 (0.09)			0
Joint Model (moderate-saliency condition)																
0.90 (0.01)	0.65 (0.03)	0.74 (0.05)	0.93 (0.01)	0.79 (0.03)	0.18 (0.07)	0.92 (0.01)	0.69 (0.03)	0.55 (0.06)	0.94 (0.01)	0.71 (0.04)	0.25 (0.07)					0
β-reparametrized Model (moderate-saliency condition)																
0.90 (0.01)	0.65 (0.03)		0.93 (0.01)	0.79 (0.03)	0.18 (0.07)	0.92 (0.01)	0.69 (0.03)		0.94 (0.01)	0.71 (0.04)	0.25 (0.07)	0.40 (0.10)	0.68 (0.06)			0
Joint Model (high-saliency condition)																
0.88 (0.01)	0.59 (0.03)	0.83 (0.03)	0.91 (0.01)	0.68 (0.03)	0.48 (0.06)	0.90 (0.01)	0.72 (0.03)	0.59 (0.06)	0.92 (0.01)	0.82 (0.03)	0.31 (0.08)					0
β-reparametrized Model (high-saliency condition)																
0.88 (0.01)	0.59 (0.03)		0.91 (0.01)	0.68 (0.03)	0.48 (0.06)	0.90 (0.01)	0.72 (0.03)		0.92 (0.01)	0.82 (0.03)	0.31 (0.08)	0.40 (0.11)	0.68 (0.08)			0

*Note.* The degree of freedom of *G*^2^ is 0

**Table 7:** Parameter estimates from the joint and reparametrized models for Experiment 2A

Context Repeated						Context Changed						New Parameters				
Ignored Repetition			Control			Ignored Repetition			Control							
*ci*	*psc*	*prr*	*ci*	*psc*	*prr*	*ci*	*psc*	*prr*	*ci*	*psc*	*prr*	β__CH_	β__REP_	α_beta	α*_ prr*_C_	*G*^*2*^
Joint Model (moderate-saliency condition)																
0.80 (0.01)	0.72 (0.02)	0.69 (0.04)	0.87 (0.01)	0.83 (0.02)	0.50 (0.08)	0.81 (0.01)	0.75 (0.02)	0.49 (0.05)	0.88 (0.01)	0.84 (0.02)	0.41 (0.08)					0
β-reparametrized Model (moderate-saliency condition)																
0.80 (0.01)	0.72 (0.02)		0.87 (0.01)	0.83 (0.02)	0.50 (0.08)	0.81 (0.01)	0.75 (0.02)		0.88 (0.01)	0.84 (0.02)	0.41 (0.08)	0.14 (0.15)	0.38 (0.13)			0
Joint Model (high-saliency condition)																
0.81 (0.01)	0.69 (0.02)	0.68 (0.04)	0.86 (0.01)	0.84 (0.02)	0.50 (0.08)	0.83 (0.01)	0.81 (0.01)	0.49 (0.07)	0.87 (0.01)	0.87 (0.02)	0.23 (0.08)					0
β-reparametrized Model (high-saliency condition)																
0.81 (0.01)	0.69 (0.02)		0.86 (0.01)	0.84 (0.02)	0.50 (0.08)	0.83 (0.01)	0.81 (0.01)		0.87 (0.01)	0.87 (0.02)	0.23 (0.08)	0.34 (0.11)	0.37 (0.13)			0
Fully Reparametrized Model (high-saliency condition)																
0.81 (0.01)	0.69 (0.02)		0.86 (0.01)	0.84 (0.02)		0.83 (0.01)	0.81 (0.01)		0.87 (0.01)	0.87 (0.02)	0.23 (0.08)		0.37 (0.13)	0.93 (0.44)	0.65 (0.12)	0

*Note.* The degree of freedom of *G*^2^ is 0

**Table 8:** Parameter estimates from the joint and reparametrized models for Experiment 2B

Context Repeated						Context Changed						New Parameters				
Ignored Repetition			Control			Ignored Repetition			Control							
*ci*	*psc*	*prr*	*ci*	*psc*	*prr*	*ci*	*psc*	*prr*	*ci*	*psc*	*prr*	β__CH_	β__REP_	α_beta	α*_ prr*_C_	*G*^*2*^
Joint Model (moderate-saliency condition)																
0.87 (0.01)	0.70 (0.03)	0.63 (0.05)	0.92 (0.02)	0.78 (0.03)	0.40 (0.08)	0.89 (0.01)	0.70 (0.03)	0.46 (0.06)	0.92 (0.01)	0.80 (0.03)	0.32 (0.08)					0
β-reparametrized Model (moderate-saliency condition)																
0.87 (0.01)	0.70 (0.03)		0.92 (0.02)	0.78 (0.03)	0.40 (0.08)	0.89 (0.01)	0.70 (0.03)		0.92 (0.01)	0.80 (0.03)	0.32 (0.08)	0.20 (0.13)	0.38 (0.12)			0
Joint Model (high-saliency condition)																
0.87 (0.01)	0.67 (0.03)	0.60 (0.05)	0.91 (0.01)	0.76 (0.03)	0.41 (0.07)	0.89 (0.01)	0.72 (0.03)	0.42 (0.06)	0.92 (0.01)	0.86 (0.03)	0.17 (0.08)					0
β-reparametrized Model (high-saliency condition)																
0.87 (0.01)	0.67 (0.03)		0.91 (0.01)	0.76 (0.03)	0.41 (0.07)	0.89 (0.01)	0.72 (0.03)		0.92 (0.01)	0.86 (0.03)	0.17 (0.08)	0.30 (0.10)	0.33 (0.12)			0
Fully Reparametrized Model (high-saliency condition)																
0.87 (0.01)	0.67 (0.03)		0.91 (0.01)	0.76 (0.03)		0.89 (0.01)	0.72 (0.03)		0.92 (0.01)	0.86 (0.03)	0.17 (0.08)		0.33 (0.12)	0.93 (0.46)	0.72 (0.11)	0

*Note.* The degree of freedom of *G*^2^ is 0

**Table 1:** Examples of stimulus configurations of different trial types for Experiments 1, 2A, and 2B

	Ignored repetition		Control		Attended repetition		Attended repetition control	
	Attended ear	Ignored ear	Attended ear	Ignored ear	Attended ear	Ignored ear	Attended ear	Ignored ear
Prime	Frog	Piano	Frog	Bell	Piano	Bell	Frog	Bell
Probe	Piano	Drum	Piano	Drum	Piano	Drum	Piano	Drum

**Table 2:** Statistical analysis results of reaction times and error rate of Experiment 1

Experiment	Repeated-measure MANOVA analysis		Results					
			Reaction Times			Error Rate		
			*F*	*p*	η_p_^2^	*F*	*p*	η_p_^2^
Experiment 1	Main Effect	Trial Type	62.60	<.001	.37	39.91	<.001	.28
		Context Relation	0.31	.58	<.01	12.53	<.01	.11
		Context Saliency	42.20	<.001	.45	11.73	<.001	.18
	Pair-wise Comparison (Context saliency)	Low vs. Moderate	0.69	>.99	.01	0.13	>.99	<.01
		Moderate vs. High	85.21	<.001	.45	23.40	<.001	.18
		Low vs. High	28.55	<.001	.21	11.23	<.01	.10

**Table 3:** MPT model analysis results of Experiment 1

Model	Restriction	Goodness-of-fit test results								
		Low-saliency			Moderate-saliency			High-saliency		
		*G*^*2*^(1)	*p*	ω	*G*^*2*^(1)	*p*	ω	*G*^*2*^(1)	*p*	ω
Baseline	*prr*_IR1_ = *prr*_C1_	13.39	<.001	.05	31.68	<.001	.08	25.15	<.001	.07
	*prr*_IR2_ = *prr*_C2_	13.70	<.001	.05	9.10	<.01	.04	6.84	<.01	.04
	*prr*_C1_ = *prr*_C2_	1.38	.24	.01	0.50	.48	.01	2.56	.11	.02
Reparametrized	*prr*_IR1_ − *prr*_C1_ = *prr*_IR2_ − *prr*_C2_	0.67	.41	.01	5.63	.02	.02	12.56	<.001	.04

*Note.* In the restriction equation of each model, 1 means context repetition, 2 means context change

**Table 4:** Statistical analysis results of reaction times and error rate of Experiments 2A and 2B

Experiment	Repeated-measure MANOVA analysis		Results					
			Reaction Times			Error Rate		
			*F*	*p*	η_p_^2^	*F*	*p*	η_p_^2^
Experiment 2A	Main Effect	Trial Type	84.85	<.001	.37	70.34	<.001	.33
		Context Relation	0.96	.33	.01	5.46	.02	.04
		Context Saliency	0.62	.43	<.01	1.20	.28	.01
Experiment 2B	Main Effect	Trial Type	115.65	<.001	.44	52.85	<.001	.27
		Context Relation	1.98	.16	.01	3.88	.05	.03
		Context Saliency	0.21	.65	<.01	0.35	.55	<.01

**Table 5:** MPT model analysis results of Experiments 2A and 2B

Experiment	Model	Restriction	Goodness-of-fit test results					
			Moderate saliency			High saliency		
			*G*^2^(1)	*p*	ω	*G*^2^(1)	*p*	ω
Experiment 2A	Baseline	*prr*_IR1_ = *prr*_C1_	4.53	.03	.03	4.40	.04	.03
		*prr*_IR2_ = *prr*_C2_	0.77	.38	.01	6.25	.01	.04
		*prr*_C1_ = *prr*_C2_	0.64	.42	.01	5.86	.02	.03
	Reparametrized	*prr*_IR1_ − *prr*_C1_ = *prr*_IR2_ − *prr*_C2_	7.82	<.01	.03	0.37	.54	.01
Experiment 2B	Baseline	*prr*_IR1_ = *prr*_C1_	5.29	.02	.04	4.49	.03	.03
		*prr*_IR2_ = *prr*_C2_	1.80	.18	.02	5.03	.02	.03
		*prr*_C1_ = *prr*_C2_	0.51	.47	.01	4.04	.04	.02
	Reparametrized	*prr*_IR1_ − *prr*_C1_ = *prr*_IR2_ − *prr*_C2_	4.66	.03	.02	0.18	.67	<.01

*Note.* In the restriction equation of each model, 1 means context repetition, 2 means context change.

## Footnotes

1 Note that the negative priming effect is considered to be rooted in multiple processes (for a review, see Frings et al., 2015), including an inhibition mechanism (Tipper, 1985) as well as an episodic memory process (Mayr & Buchner, 2006; Neill & Valdes, 1992). For the purposes of the present paper, we exclusively focus on the latter.

2 By generating the ignored repetition and attended repetition trials in the described way, each control trial would have occurred twice, once as a control for an ignored repetition trial and once as a control for an attended repetition trial. To avoid this confounding, the ignored repetition and attended repetition trials and their respective control trials were systematically assigned to two basic sets (referred to as Set 1 and Set 2). In each basic set, none of the control trials was repeated. Participants were randomly assigned to one of these two basic sets. For more details of Set 1 and Set 2, please see Mayr and Buchner (2006).

3 For the abbreviation of each parameter, *ci* means *correct identification*, *psc* means *probe stimulus confusion*, *prr* means *prime-response retrieval*.

4 We thank an anonymous reviewer for this idea that similarity breeds perceptual grouping, which may result in a compound of the distractor stimulus and the response in the so-called configural binding.

## References

[CR1] Biggs AT, Kreager RD, Gibson BS, Villano M, Crowell CR (2012). Semantic and affective salience: The role of meaning and preference in attentional capture and disengagement. Journal of Experimental Psychology: Human Perception and Performance.

[CR2] Bouton ME (1984). Differential control by context in the inflation and reinstatement paradigms. Journal of Experimental Psychology: Animal Behavior Processes.

[CR3] Bouton ME, Mesquita B, Barrett LF, Smith ER (2010). The multiple forms of "context" in associative learning theory. *The mind in context*.

[CR4] Bouton ME, King DA (1986). Effect of context on performance to conditioned-stimuli with mixed histories of reinforcement and nonreinforcement. Journal of Experimental Psychology: Animal Behavior Processes.

[CR5] Bouton ME, Todd TP (2014). A fundamental role for context in instrumental learning and extinction. Behavioural Processes.

[CR6] Chao HF (2009). Revisiting the prime–probe contextual similarity effect on negative priming: The impact of cue variability. European Journal of Cognitive Psychology.

[CR7] Chao HF, Yeh YY (2008). Attentional demand and memory retrieval in negative priming. Psychological Research.

[CR8] Deutsche Gesellschaft für Psychologie. (2016). *Berufsethische Richtlinien*. https://www.bdp-verband.de/binaries/content/assets/beruf/ber-foederation-2016.pdf

[CR9] Dutzi IB, Hommel B (2009). The microgenesis of action-effect binding. Psychological Research-Psychologische Forschung.

[CR10] Fanselow MS (1980). Conditional and unconditional components of post-shock freezing. Pavlovian Journal of Biological Science.

[CR11] Faul F, Erdfelder E, Buchner A, Lang AG (2009). Statistical power analyses using G* Power 3.1: Tests for correlation and regression analyses. Behavior Research Methods.

[CR12] Feldman J (2013). The neural binding problem(s). Cognitive Neurodynamics.

[CR13] Frings C, Rothermund K (2017). How perception guides action: Figure–ground segmentation modulates integration of context features into SR episodes. Journal of Experimental Psychology: Learning, Memory, and Cognition.

[CR14] Frings C, Schneider KK, Fox E (2015). The negative priming paradigm: An update and implications for selective attention. Psychonomic Bulletin & Review.

[CR15] Frings C, Koch I, Moeller B (2017). How the mind shapes action: Offline-contexts modulate involuntary episodic retrieval. Attention, Perception, & Psychophysics.

[CR16] Frings C, Hommel B, Koch I, Rothermund K, Dignath D, Giesen C, Kiesel A, Kunde W, Mayr S, Moeller B, Moller M, Pfister R, Philipp AM (2020). Binding and retrieval in action control (BRAC). Trends in Cognitive Sciences.

[CR17] Goddard MJ, Holland PC (1996). Type of feature affects transfer in operant serial feature-positive discriminations. Animal Learning & Behavior.

[CR18] Hoffman DD, Singh M (1997). Salience of visual parts. Cognition.

[CR19] Holland PC (1989). Occasion setting with simultaneous compounds in rats. Journal of Experimental Psychology: Animal Behavior Processes.

[CR20] Holland PC, Haas ML (1993). The effects of target salience in operant feature positive discriminations. Learning and Motivation.

[CR21] Holm S (1979). A simple sequentially rejective multiple test procedure. Scandinavian Journal of Statistics.

[CR22] Hommel B (1998). Event files: Evidence for automatic integration of stimulus–response episodes. Visual Cognition.

[CR23] Hommel B (2004). Event files: Feature binding in and across perception and action. Trends in Cognitive Sciences.

[CR24] Honey RC, Iordanova MD, Good M (2014). Associative structures in animal learning: Dissociating elemental and configural processes. Neurobiology of Learning and Memory.

[CR25] Hu X, Batchelder WH (1994). The statistical-analysis of general processing tree models with the EM algorithm. Psychometrika.

[CR26] Iordanova MD, Burnett DJ, Aggleton JP, Good M, Honey RC (2009). The role of the hippocampus in mnemonic integration and retrieval: Complementary evidence from lesion and inactivation studies. European Journal of Neuroscience.

[CR27] Kahneman D, Treisman A, Gibbs BJ (1992). The reviewing of object files: Object-specific integration of information. Cognitive Psychology.

[CR28] Kayser C, Petkov CI, Lippert M, Logothetis NK (2005). Mechanisms for allocating auditory attention: An auditory saliency map. Current Biology.

[CR29] Knapp BR, Batchelder WH (2004). Representing parametric order constraints in multi-trial applications of multinomial processing tree models. Journal of Mathematical Psychology.

[CR30] Logan GD (1988). Toward an instance theory of automatization. Psychological Review.

[CR31] Mayr S, Buchner A (2006). Evidence for episodic retrieval of inadequate prime responses in auditory negative priming. Journal of Experimental Psychology: Human Perception and Performance.

[CR32] Mayr S, Möller M, Buchner A (2018). Contextual modulation of prime response retrieval processes: Evidence from auditory negative priming. Attention, Perception, & Psychophysics.

[CR33] Moeller B, Frings C, Pfister R (2016). The structure of distractor-response bindings: Conditions for configural and elemental integration. Journal of Experimental Psychology: Human Perception and Performance.

[CR34] Moshagen M (2010). multiTree: A computer program for the analysis of multinomial processing tree models. Behavior Research Methods.

[CR35] Neill WT (1977). Inhibitory and facilitatory processes in selective attention. Journal of Experimental Psychology: Human Perception and Performance.

[CR36] Neill WT, Valdes LA (1992). Persistence of negative priming: Steady-state or decay. Journal of Experimental Psychology: Learning Memory and Cognition.

[CR37] NIOSH. (2016). NIOSH Sound Level Meter (Version 1.2.2) [Mobile app]. https://apps.apple.com/us/app/niosh-sound-level-meter/id1096545820. Accessed 30 Oct 2019

[CR38] Niu YQ, Todd RM, Anderson AK (2012). Affective salience can reverse the effects of stimulus-driven salience on eye movements in complex scenes. Frontiers in Psychology.

[CR39] Ogawa T, Suzuki N (2004). On the saliency of negative stimuli: Evidence from attentional blink. Japanese Psychological Research.

[CR40] Pearce JM, Bouton ME (2001). Theories of associative learning in animals. Annual Review of Psychology.

[CR41] Peirce JW, Gray JR, Simpson S, MacAskill MR, Höchenberger R, Sogo H, Kastman E, Lindeløv J (2019). PsychoPy2: Experiments in behavior made easy. Behavior Research Methods.

[CR42] Rescorla RA, Wagner AR, Black AH, Prokasy WE (1971). A theory of pavlovian conditioning: Variations in the effectiveness of reinforcement and nonreinforcement. *Classical conditioning II: Current theory and research*.

[CR43] Rothermund K, Wentura D, De Houwer J (2005). Retrieval of incidental stimulus–response associations as a source of negative priming. Journal of Experimental Psychology: Learning Memory and Cognition.

[CR44] Seymour K, Clifford CW, Logothetis NK, Bartels A (2009). The coding of color, motion, and their conjunction in the human visual cortex. Current Biology.

[CR45] Shanks DR, Charles D, Darby RJ, Azmi A (1998). Configural processes in human associative learning. Journal of Experimental Psychology: Learning Memory and Cognition.

[CR46] Smith SM, Vela E (2001). Environmental context-dependent memory: A review and meta-analysis. Psychonomic Bulletin & Review.

[CR47] Stecker GC, Harrington IA, Middlebrooks JC (2005). Location coding by opponent neural populations in the auditory cortex. PLOS Biololgy.

[CR48] Sui J, He X, Humphreys GW (2012). Perceptual effects of social salience: Evidence from self-prioritization effects on perceptual matching. Journal of Experimental Psychology: Human Perception and Performance.

[CR49] Tipper SP (1985). The negative priming effect—Inhibitory priming by ignored objects. Quarterly Journal of Experimental Psychology.

[CR50] Todd TP, DeAngeli NE, Jiang MY, Bucci DJ (2017). Retrograde amnesia of contextual fear conditioning: Evidence for retrosplenial cortex involvement in configural processing. Behavioral Neuroscience.

[CR51] Treisman A (1996). The binding problem. Current Opinion in Neurobiology.

[CR52] Wagemans J, Elder JH, Kubovy M, Palmer SE, Peterson MA, Singh M, von der Heydt R (2012). A century of gestalt psychology in visual perception: I. Perceptual grouping and figure–ground organization. Psychological Bulletin.

